# Epoxy-Encapsulated ZnO–MWCNT Hybrid Nanocomposites with Enhanced Thermoelectric Performance for Low-Grade Heat-to-Power Conversion

**DOI:** 10.3390/polym15234540

**Published:** 2023-11-26

**Authors:** Margarita Volkova, Raitis Sondors, Elmars Spalva, Lasma Bugovecka, Artis Kons, Raimonds Meija, Jana Andzane

**Affiliations:** 1 Institute of Chemical Physics, University of Latvia, Raina Blvd. 19, LV-1586 Riga, Latvia; 2 3D Strong Ltd., Instituta Str. 36–17, LV-2130 Ulbroka, Latvia; 3 Faculty of Chemistry, University of Latvia, Raina Blvd. 19, LV-1586 Riga, Latvia

**Keywords:** zinc oxide–carbon nanotube nanocomposite, hybrid network, epoxy-based thermoelectric nanocomposite, ZnO nanostructured network, domestic waste heat conversion, multiwall carbon nanotubes

## Abstract

This work is devoted to the development of epoxy-encapsulated zinc oxide-multiwalled carbon nanotubes (ZnO–MWCNT) hybrid nanostructured composites and the investigation of their thermoelectric performance in relation to the content of MWCNTs in the composite. For the preparation of nanocomposites, self-assembling Zn nanostructured networks were coated with a layer of dispersed MWCNTs and subjected to thermal oxidation. The resulting ZnO–MWCNT hybrid nanostructured networks were encapsulated in commercially available epoxy adhesive. It was found that encapsulation of ZnO–MWCNT hybrid networks in epoxy adhesive resulted in a simultaneous decrease in their electrical resistance by a factor of 20–60 and an increase in the Seebeck coefficient by a factor of 3–15, depending on the MWCNT content. As a result, the thermoelectric power factor of the epoxy-encapsulated ZnO–MWCNTs hybrid networks exceeded that of non-encapsulated networks by more than 3–4 orders of magnitude. This effect was attributed to the ZnO–epoxy interface’s unique properties and to the MWCNTs’ contribution. The processes underlying such a significant improvement of the properties of ZnO–MWCNT hybrid nanostructured networks after encapsulation in epoxy adhesive are discussed. In addition, a two-leg thermoelectric generator composed of epoxy-encapsulated ZnO–MWCNT hybrid nanocomposite as n-type leg and polydimethylsiloxane-encapsulated CuO–MWCNT hybrid nanocomposite as p-type leg characterized at room temperatures showed better performance at temperature difference 30 °C compared with the similar devices, thus proving the potential of the developed nanocomposites for applications in domestic waste heat conversion devices.

## 1. Introduction and background

Zinc oxide (ZnO) is an n-type semiconductor that has good biocompatibility and high mechanical, thermal, and chemical stability [1,2]. It is also intensively studied for a wide variety of applications, including but not limited to light emitters [3], sensors [4,5,6,7], photovoltaics [8], photocatalysis [9], field effect transistors [10], and power nanogenerators based on piezoelectric [11] and/or thermoelectric [12,13] effects. In most applications, ZnO is used in nanostructured form to enhance its properties due to high surface-to-volume ratio [14,15]. Among different synthesis methods, the thermal oxidation of Zn to obtain ZnO nanostructures is one of the most favorable as it does not require high-tech equipment, harmful chemicals, or extremely high synthesis temperatures and results in a high yield of ZnO nanowires. Typically, the process of thermal oxidation of Zn occurs in the air or oxygen environment at temperatures ranging from 300 to 600 °C [16,17,18,19,20,21,22,23,24,25].

ZnO-based materials and composites are also studied for their thermoelectric performance as the metal oxides are considered low-cost and environmentally friendly alternatives to toxic, rare, and costly conventional thermoelectric materials such as lead, bismuth, and antimony chalcogenides [26]. The efficiency of the thermoelectric materials is characterized by a dimensionless figure of merit ZT = S^2^·σ·T·κ^−1^, where S is the Seebeck coefficient, σ is the electrical conductivity, T is the absolute temperature, and κ is the thermal conductivity. (S^2^·σ) is traditionally referred to as the material’s thermoelectric power factor (PF). Thus, a high Seebeck coefficient, which depends on charge carrier concentration [27], is crucial for increasing the ZT of thermoelectric material and reducing κ without affecting σ. To date, most of the reported Seebeck coefficients for ZnO-based thermoelectric materials (thin films, ZnO powder-based ceramics, nanowire networks) ranged from ~−100 to ~−380 μV·K^−1^ [28,29,30,31]. However, the relatively low electrical conductivity of ZnO-based materials (typically in the range of 1–25 S·cm^–1^ or below that value) is an obstacle to applying these materials in thermoelectrics. It has been reported that electrical conductivity and charge carrier separation efficiency in ZnO can be improved by fabricating hybrid materials joining ZnO with carbon nanotubes (CNT) [32,33,34,35,36,37,38,39]. Most of these works report properties of the materials at significantly elevated temperatures and/or measured under an inert environment, thus not focusing on the practical applications of the developed materials in the form of polymer-based composites, where a ZnO-based nanostructured material is used as a filler or is encapsulated in the protective polymer layer. Only a limited number of reports show the performance of ZnO-based materials in thermoelectric generators [12,34,40]. For example, Klochko et al. [40] reported a flexible thermoelectric generator consisting of ZnO thin films with S~−97 μV·K^−1^ deposited by atomic layer deposition on flexible polyimide substrates. However, the research of polymer- or epoxy-based composites containing nanostructured ZnO filler is up to date and focused on the investigation of their mechanical, thermal, electrical, and optical properties for applications not related to thermoelectrics [41,42,43]. Recently, our group showed that encapsulation of self-assembling ZnO nanowire networks in a commercially available epoxy adhesive results in a dramatic increase in PF of the epoxy–ZnO system by a few orders of magnitude [25]. This effect was attributed to the unique properties of the ZnO–epoxy interface, facilitating the increase in the electrical conductance of the ZnO nanowire network simultaneously with the increase in its Seebeck coefficient.

The present work is focused on developing epoxy-based ZnO–MWCNT hybrid nanocomposites and investigating their thermoelectric properties in relation to the MWCNT content in the ZnO–MWCNT hybrid nanostructured networks. The ZnO–MWCNT hybrid nanostructured networks were fabricated by thermal oxidation of Zn–MWCNT networks, where self-assembling in a sponge-like structure, Zn nanostructures were coated with a layer of dispersed MWCNTs. After the oxidation, ZnO–MWCNT hybrid networks containing different amounts of embedded multiwalled carbon nanotubes were encapsulated in epoxy adhesive. To the best of our knowledge, the thermoelectric properties of epoxy-encapsulated ZnO–MWCNT hybrid nanostructured composites were investigated for the first time.

## 2. Materials and Methods

### 2.1. Synthesis of Metallic Zn Nanostructured Networks

Zinc foil (99.95%, thickness 50 μm, Goodfellow GmbH, Hamburg, Germany) was cleaned in acetone and isopropanol cut in pieces of ~0.3 g and used as Zn source material. Pre-cleaned Zn foil was placed into the center of the hot zone of a horizontal single-zone quartz furnace tube (OTF-1200X, MTI Corp., Richmond, CA, USA). The glass substrates (Avantor, Radnor, PA, USA) were placed into the area of the furnace tube, where the temperature during the deposition process will reach 300–350 °C. For the evaporation of Zn source material, the furnace tube was flushed for a few minutes with N_2_, pumped down to a basic pressure of 0.2 Torr, and sealed. The sealed tube was heated to the temperature of 450 °C in the center of the tube at a rate of 15 °C·min^−1^ and kept at this temperature for the next 30 min, followed by natural cooling down to room temperature.

### 2.2. Synthesis and Spray-Coating of MWCNTs

MWCNTs were synthesized using the spray-assisted chemical vapor deposition method described elsewhere [44]. The synthesis occurred in an inert (argon) atmosphere under atmospheric pressure at a flow rate of 20 mm·s^–1^ using 2 wt.% of ferrocene (used for forming catalyst nanoparticles during the synthesis) dissolved in toluene for one synthesis cycle. The synthesis was carried out for 60 min at a temperature of 800 °C. For the spray coating, MWCNTs were sonicated with isopropyl alcohol in an ultrasonic bath (Sonorex RK 100, BANDELIN electronic GmbH & Co. KG, Berlin, Germany) to obtain a solution with MWCNT concentration 0.1 mg·mL^–1^. The solution was sprayed over the prefabricated Zn nanostructured networks to obtain Zn–MWCNT hybrid networks with 0.125, 0.25, and 0.5 wt.% of MWCNTs.

### 2.3. Oxidation of Zn–MWCNT Nanostructured Networks

Glass substrates with the Zn–MWCNT nanostructured networks were placed in the center of the hot zone of the furnace tube (OTF-1200X, MTI Corp., Richmond, CA, USA). The furnace tube remained open to the ambient air. The furnace was heated to 450 °C at a rate of ~15 °C·min^–1^ and kept at this temperature for the next 30 min, followed by natural cooling to the room temperature.

### 2.4. Encapsulation of ZnO Nanowire Network with Epoxy Adhesive

Epoxy adhesive (Henkel AG & Co. KGaA, Dusseldorf, Germany) was drop-casted on the surface of the ZnO–MWCNT nanostructured network sample, followed by its spread throughout the network due to the capillary forces and dried under ambient conditions for the next 24 h.

### 2.5. Morphological and Structural Characterization

The structure and morphology of prepared samples were inspected using a field-emission scanning electron microscope (SEM) Hitachi S-4800 (Hitachi Ltd., Tokyo, Japan). Powder X-ray diffraction (XRD) measurements were performed on a Bruker D8 Discover diffractometer (Bruker Corp., Billerica, MA, USA) using copper radiation source (Cu Kα = 1.54180 Å) with Bragg−Brentano geometry and a LynxEye (1D) detector. The divergence and antiscattering slits were set at 0.6 mm and 8 mm, respectively. The patterns were recorded from 10° to 70° on the 2θ scale, using a scan speed of 0.5 s/0.02°. For the identification of the diffraction peaks, the ICDD database PDF-2/Release 2021 was used (Ref. cards PDF 01-078-9363 Zn, PDF 01-075-6445 ZnO, and PDF 01-086-3978 ZnO).

### 2.6. Electrical and Thermoelectric Characterization

For electrical and thermoelectric measurements, electrical contacts were fabricated at the sides of the samples using silver paint (Ted Pella, Inc., Redding, CA, USA) and copper wires (cross-section 0.2 mm^2^) bonded to it. The distance between the electrodes was 10 mm, and the sample width was 10 mm. Current–voltage curves of the sample were measured by a Keithley 6487 picoammeter/voltage source (Keithley Instruments, Cleveland, OH, USA). Seebeck coefficient measurements were carried out at room temperature under ambient conditions using a lab-made device reported elsewhere [45] and calibrated by NIST Standard Reference material 3451 (NIST, Gaithersburg, MD, USA) for low-temperature Seebeck coefficient. Thermoelectric voltage, generated by the ZnO–MWCNT hybrid networks, was registered by HP 34401A multimeter (Hewlett-Packard Company, Palo Alto, CA, USA) and custom software for automatic data recording at different temperature gradients applied to the sample (20 measurements for each temperature difference). The slope of the thermoelectric voltage vs. temperature difference curve gave the averaged Seebeck coefficient values. The temperature gradient between the sample sides did not exceed 10% of the absolute temperature at which the measurements were performed. The ± error bars were estimated as standard deviation (SD) from the measurements for each temperature difference.

### 2.7. Bending Tests

Bending tests of the samples were performed in a 2-point configuration using a custom experimental setup, allowing simultaneous bending of the sample down to a 5 mm radius and measuring its electrical resistance using a Keithley 6430 Sub-Femtoamp remote source meter (Keithley Instruments, Cleveland, OH, USA), combined with a custom software. The resistance of the samples was measured at a constant voltage of 0.1 V applied to the sample. The ± error bars were estimated as SD from 3 to 5 measurements for each point.

### 2.8. Assembly of the Two-Leg Thermoelectric Generator

The two-leg thermoelectric generator (TEG) prototype was constructed by sequentially stacking insulation foam layers and nanostructure films. Initially, using low-expansion polyurethane (PU) foam (Tytan Professional, Wrocław, Poland), a base foam layer of dimensions 1 by 1 cm by 0.5 mm height was formed and left to slightly solidify for 20 min, such that the layer is still malleable yet holds its shape. Then, the epoxy-encapsulated ZnO–MWCNT film with pre-fabricated contacts was set on top of the PU layer, with the epoxy facing downwards. Subsequently, another foam layer was formed, this time of 1 cm thickness, and it was once again left for 20 min to solidify. After that, the encapsulated polydimethylsiloxane (PDMS) CuO–MWCNT film was set on top, with PDMS facing upwards. The finishing layer of foam was formed on top of the encapsulated CuO–MWCNT film with the same dimensions as the first layer, after which the prototype was left to fully cure for 24 h as per the minimal requirements of the manufacturer. Post-curing, excess material was carefully trimmed to align with the dimensions of the TE films, ensuring flat sides and direct contact between the TE films and heating elements for subsequent characterization. The final step was to solder the wires of one side of the films together.

## 3. Results and Discussion

### 3.1. Fabrication and Morphology of ZnO–MWCNT Hybrid Networks

**Figure 1:**
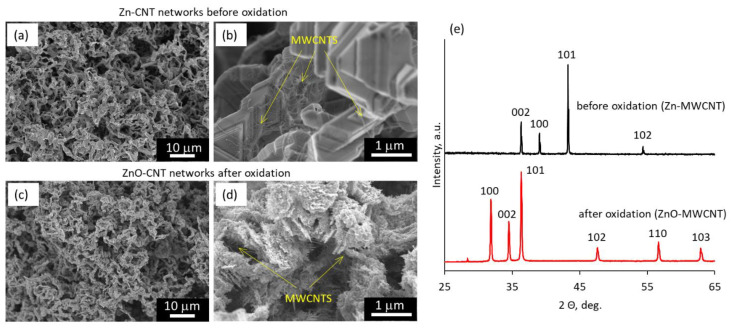
Scanning electron microscope images of (**a**,**b**) Zn nanowire networks coated with MWCNT layer (total mass of the deposited MWCNTs 1 mg) and (**c**,**d**) the same network after the thermal oxidation process; (**e**) X-ray diffraction patterns of the Zn–MWCNT network before (black curve) and after (red curve) the thermal oxidation process.

To fabricate ZnO–MWCNT hybrid networks, pre-synthesized Zn nanostructure networks were coated by a layer of MWCNTs using the spray-coating method (Figure 1a,b) and oxidized as described in section Materials and Methods. A typical Zn nanostructured network consists of stacked Zn nanostructures/nanoplates of sharp-corner triangular, hexagonal, or trapezoidal shape (Figure 1b) with thicknesses of a few tens of nm and surface areas varying from ~0.5 to ~2 mm^2^, interconnected in a sponge-like structure. The mass fraction of the MWCNTs in the prepared Zn–CNT samples was 0.125, 0.25, and 0.5 wt.%. As is seen in Figure 1a, MWCNTs formed a random network over the Zn nanostructures.

The XRD patterns obtained for the Zn–MWCNT network showed the presence of the prominent crystallographic peaks related to the (002), (100), (101), and (102) crystallographic planes of hexagonal phase Zn (Figure 1e, black curve). The peak intensities corresponded to the reference Zn metal, indicating no preferential growth direction/orientation of Zn nanostructures. The peaks related to the ZnO phase were not detected, proving that the MWCNTs were deposited on a metallic Zn nanostructured network not coated with the oxide layer.

After the oxidation process, the morphology of the oxidized Zn–MWCNT nanostructures forming the network changed from the sharp-sided shape (Figure 1a,b) to the nanoflakes of irregular shape, embedding the MWCNTs (Figure 1c,d). The XRD patterns obtained for the oxidized Zn–MWCNT nanostructured network showed diffraction peaks corresponding to (100), (002), (101), (102), (110), and (103) crystallographic planes of wurtzite ZnO (Figure 1e, red curve). The absence of the diffraction peaks corresponding to metallic Zn indicated its complete transformation to ZnO. Further in the text, the oxidized Zn–MWCNT nanostructured networks will be referred to as ZnO–MWCNT hybrid networks. No significant difference in morphology was observed for the ZnO–MWCNT hybrid networks containing different wt.% of MWCNTs.

### 3.2. Electrical and Thermoelectric Properties of ZnO–MWCNTs Hybrid Networks

**Figure 2:**
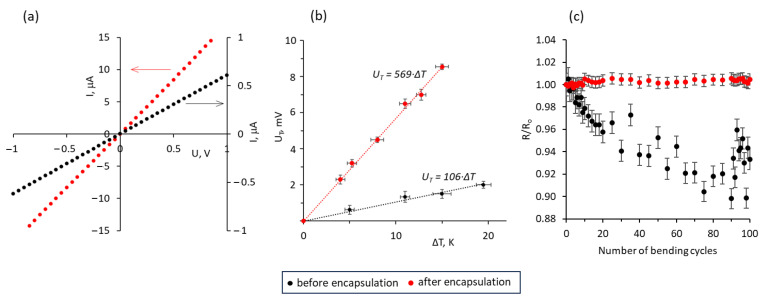
(**a**) Current–voltage curves of the ZnO–MWCNT hybrid network containing 0.25 wt.% of MWCNTs before (black dots, secondary scale) and after (red dots, primary scale) encapsulation in epoxy adhesive; (**b**) thermally generated by the ZnO–MWCNT hybrid network voltage vs. temperature difference applied between its sides before (black dots) and after (red dots) encapsulation; (**c**) ratios of the resistance of the bent sample (R) to its initial resistance (R_0_) vs. number of bending cycles down to 5 mm radius for ZnO–MWCNT hybrid network with 0.5 wt.% of MWCNTs before (black dots) and after (red dots) encapsulation.

To preserve the structure of ZnO–MWCNT hybrid networks, they were encapsulated in commercially available epoxy adhesive, which has been previously found to improve the electrical and thermoelectric properties of ZnO nanostructures. Encapsulation of the ZnO–MWCNT hybrid networks resulted in a significant reduction of their electrical resistance and simultaneously in great improvement of the Seebeck coefficient. The representative current–voltage (I-V) curves and thermally generated voltage (U_T_) of the ZnO–MWCNT hybrid network containing 0.25 wt% of MWCNTs before (black dots) and after (red dots) the encapsulation is shown in Figure 2a,b, respectively.

The current–voltage curves of the ZnO–MWCNT hybrid networks before and after encapsulation in the epoxy adhesive showed linear behavior, indicating good electrical contacts between the network components, which were not destroyed by the encapsulation process (Figure 2a, Figures S1–S3 in Supplementary Material). Before the encapsulation, the resistance of the ZnO and ZnO–MWCNT networks varied in the range of 10^5^–10^6^ Ω and decreased after the encapsulation of the samples by ~18–60 times, depending on the sample (Table 1). The preservation of the established electrical connections in the ZnO–MWCNT hybrid network during the synthesis process was supported by the bending tests (Figure 2c). The encapsulated networks showed the stability of the resistance during the 100 consecutive bending cycles down to the 5 mm radius with fluctuations within 1% from the initial resistance of the non-bent sample (Figure 2c, red dots). In comparison, non-encapsulated networks showed a gradual decrease in the resistance during the first 80 bending cycles, reaching 90% of the initial resistance of the non-bent sample, followed by its increase during the last 20 cycles by ~7% (Figure 2c, black dots). This instability of the resistance of the non-encapsulated sample may be related to the disruption of the existing electrical connections and the formation of new ones during the bending. In this case, the general tendency of the resistance decrease may indicate the separation of MWCNTs from the ZnO nanostructures and the formation of more electrical connections between the MWCNTs.

**Figure 3:**
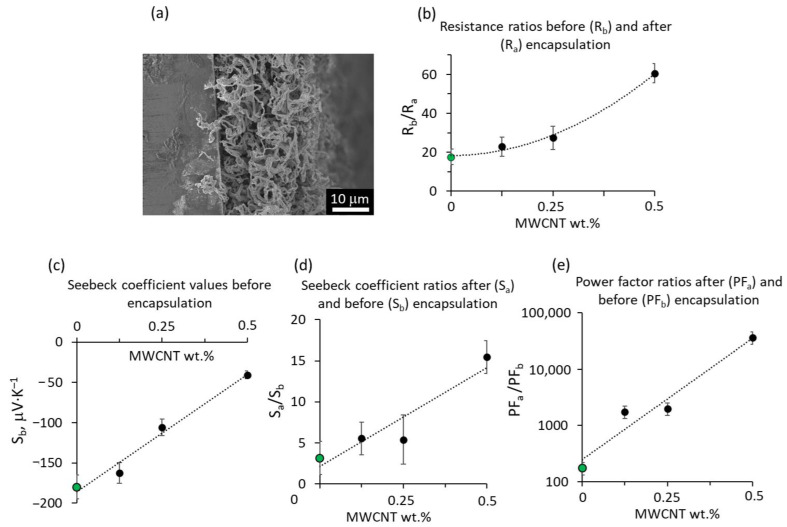
(**a**) Scanning electron microscope image of a cross-section of a ZnO-CNT hybrid composite; (**b**–**d**) relative comparison of resistance of ZnO network (green dots) and ZnO-CNT hybrid nanostructured composites (black dots) before and after encapsulation in epoxy adhesive vs. MWCNT content in the composite; (**c**) Seebeck coefficient of ZnO–MWCNT hybrid networks before encapsulation in epoxy adhesive vs. MWCNT content; (**d**,**e**) relative comparison of the (**d**) Seebeck coefficient; and (**e**) power factor of ZnO network (green dots) and ZnO-CNT hybrid nanostructured composites (black dots) before and after encapsulation in epoxy adhesive vs. MWCNT content in the composite.

It should be noted that the absolute values of the resistance of different samples cannot be directly compared due to the complex structure of the nanostructured, nanoporous samples, having varying thicknesses and active cross-section areas (Figure 3a). Thus, for further analysis, the relative comparison of the samples before and after encapsulation was performed. The magnitude of the resistance decrease showed a tendency for the power-law to increase with the increase in MWCNT content in the ZnO–MWCNT hybrid networks (Figure 3b).

The decrease in the resistance of the bare ZnO nanowire network after the encapsulation in epoxy adhesive was explained by the interaction of ZnO nanoparticles with epoxy, involving the formation of an electrically conductive dual nanolayer interface between the ZnO and epoxy adhesive. The first nanolayer is firmly attached to each other, and the ZnO surface epoxy segments due to the formation of hydrogen bonds between the epoxide groups and free hydroxyl (OH) groups formed due to water chemisorption on the ZnO surface. Presumably, the high number of hydrogen bonds mitigates the interface polarization effect and promotes the transport of electrons generated due to the chemisorption of water molecules, thus making this layer electrically conductive [43,46,47]. However, the power-law decrease in the resistance of the ZnO–MWCNT hybrid nanocomposites with the increase in MWCNT content in them the result of additional to the epoxy contribution of MWCNTs to the total conductance of the nanocomposite. As can be seen from Figure 1 d, after the thermal oxidation of the Zn–MWCNT network, some proportion of MWCNTs remained partially embedded within ZnO nanostructures and partially outside the ZnO nanostructures. Presumably, during the encapsulation process, when the epoxy adhesive fills the pores within the ZnO–MWCNT nanostructured network, and during the further curing of the epoxy adhesive, the MWCNTs formed an electrically conductive network within the epoxy. This hypothesis is in line with the previously reported sharp conductivity increase that was observed for the epoxy-CNT composites starting from 0.05 wt.% of MWCNTs by other research groups [48]. In addition, the charge transfer from the ZnO to MWCNT may also facilitate enhanced conductance through the established MWCNT network [49].

The inclusion of MWCNTs in the ZnO–MWCNT hybrid nanostructured networks (before encapsulation in epoxy adhesive) resulted in a linear decrease in their Seebeck coefficient with the increase in the MWCNT content from −180 μV·K^–1^ to −40 μV·K^–1^ (Table 2, Figure 3c). Most likely, the decrease in the Seebeck coefficient is due to the contribution of the MWCNT network to the total Seebeck coefficient of the ZnO–MWCNT composite as the as-grown, not doped MWCNTs are known to be p-type [44], which is the opposite to ZnO being the n-type semiconductor. It should be noted that the Seebeck coefficient values obtained for the ZnO–MWCNT hybrid networks were comparable with the Seebeck coefficient values reported for the other ZnO- and CNT-based composite materials (Table 2).

However, the encapsulation of ZnO–MWCNT hybrid networks in epoxy adhesive resulted in a significant increase in their Seebeck coefficient by a factor of 3–15 (Table 2, Figure 3d). The lowest relative increase in the Seebeck coefficient was observed for the ZnO nanostructured network without CNTs. In contrast, the highest relative increase was observed for the ZnO–MWCNT hybrid network containing 0.5 wt.% of MWCNTs. At the same time, the absolute values of the Seebeck coefficient of the encapsulated ZnO and ZnO–MWCNT nanostructured networks varied in the range from 570 up to 900 μV·K^–1^ (Table 2), showing no direct correlation with the MWCNT content in the nanocomposite. This indicates that in the encapsulated ZnO–MWCNT hybrid networks, the dominating contribution to the Seebeck coefficient is provided by the ZnO nanostructures, and the opposing contribution of the p-type MWCNTs is negligible compared to the absolute Seebeck coefficient values ensured by the n-type ZnO nanostructures. Such a significant increase in the Seebeck coefficient in ZnO–epoxy systems compared to ZnO may be related to the contribution of the second layer in the double-layer interface formed between the ZnO and epoxy adhesive, consisting of loose epoxy segments, facilitating the easy transfer of charge carriers and simultaneously having a high probability of defect acting as charge trapping sites [47,51]. Thus, the presence of the second ZnO–epoxy interface layer may significantly impact the charge carrier concentration in the ZnO–epoxy system, making it optimal for reaching high Seebeck coefficient values. Comparison of the Seebeck coefficient of the epoxy-encapsulated ZnO–MWCNT hybrid networks with other similar work was difficult due to the minimal number of reported similar epoxy adhesive-based composites for thermoelectric applications. However, it was found that the Seebeck coefficient of epoxy-encapsulated ZnO–MWCNT hybrid networks with MWCNT content 0.5 wt.% exceeds the Seebeck coefficient showed by the epoxy resin-based compound prepared by mixing of 8 wt.% of MWCNTs with TiO_2_ nanoparticles by ~25 times (Table 2). These results indicate that, presumably, such a high Seebeck coefficient of the epoxy-encapsulated ZnO and ZnO–MWCNT nanostructured composites was due to the unique properties of the interface formed between ZnO and epoxy adhesive.

Assuming that the changes in the geometry in terms of length, width, and the active electrically conductive cross-section area of the ZnO and ZnO–MWCNT hybrid networks were insignificant, the ratios of the power factor of the samples after and before encapsulation (PF_a_ and PF_b_) will be proportional to the ratio of the squares of their Seebeck coefficients (S_a_ and S_b_) divided by the ratio of their resistances (R_a_ and R_b_) as $\left(\frac{{PF}_{a}}{{PF}_{b}}=\frac{S_{a}^{2}R_{b}}{S_{b}^{2}R_{a}}\right).$ Such relative comparison of the PF of the ZnO and ZnO–MWCNT hybrid networks before and after encapsulation in epoxy adhesive showed that the PF of the encapsulated ZnO–MWCNT hybrid networks were by 3–4 orders of magnitude higher compared to the non-encapsulated and by an order of magnitude higher compared to the encapsulated ZnO nanostructured network (Figure 3e). The dramatic increase in the PF of the encapsulated samples compared to the non-encapsulated ones is mostly due to the increase in the Seebeck coefficient, which was especially pronounced for the samples containing higher amounts of MWCNTs (Table 2). In turn, the higher PF of encapsulated ZnO–MWCNT hybrid networks compared to the encapsulated ZnO network was due to the additional contribution of the MWCNTs to the decrease in the resistance of the encapsulated networks (Figure 3b, Table 1).

**Figure 4:**
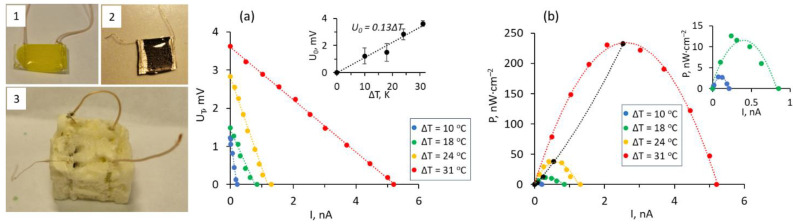
(1)–(3) photo images of (1) epoxy-encapsulated ZnO–MWCNT hybrid networks containing 0.5 wt.% of MWCNTs; (2) PDMS-encapsulated CuO–MWCNT hybrid composite containing 0.9 wt.% of MWCNTs; and (3) two-leg thermoelectric generator, where encapsulated ZnO–MWCNT and CuO–MWCNT nanocomposites are embedded in thermally insulating foam; (**a**) thermally generated by the TEG voltage vs. electrical current under different external loads connected in series and under temperature differences applied between the sides of the TEG; inset – thermally generated by the TEG open-circuit voltage vs. temperature difference applied to the TEG; (**b**) calculated TEG output power vs. output current under different temperature differences applied between the sides of the TEG; inset—close view of the output power vs. output current curves obtained for the temperature differences 10 °C and 20 °C.

To evaluate the potential of the presented epoxy-encapsulated ZnO–MWCNT nanocomposites for application in waste-to-power conversion applications, a simple two-leg thermoelectric generator (TEG) was fabricated by embedding the epoxy-encapsulated n-type ZnO–MWCNT hybrid nanocomposite containing 0.25 wt.% (Figure 4 (1)) and developed previously by our group p-type CuO–MWCNT nanocomposite encapsulated in polydimethylsiloxane (PDMS) [52] (Figure 4 (2)) in commercially available foam for the thermal insulation (Figure 4 (3)).

The performance of TEG was characterized by measuring thermally generated voltage U_T_ at room temperature under different external loads connected in series and under temperature differences of 10–31 °C applied between the sides of the TEG (Figure 4a). TEG showed nearly linear performance that can be described by a function U_o_ = 0.13ΔT (Figure 4a, inset). However, unequal slopes of U_T_ vs. ΔT curves (Figure 4a) demonstrated that the TEG performance is unstable at low-temperature differences and becomes more reliable at temperature differences at and above ~30 °C (Figure 4a, red curve). The calculated output power P vs. output current showed the power-law dependence of the maximal power generated at different temperatures with the maximal reached P value of ~230 nW·cm^–1^ at ΔT = 31 °C (Figure 4b). The experimentally observed performance of this TEG composed of epoxy-encapsulated n-type ZnO–MWCNT and PDMS-encapsulated p-type CuO–MWCNT legs was lower than would be expected from the Seebeck coefficient values of the separate legs being within the range of 500–600 μV·K^–1^, which is most likely related to the significant energy dissipation at the electrodes and connections between the legs, as well as probably to the unwanted Joule heating of the TEG components. These results outline the further actions for the improvement of the performance of such TEGs, which should be focused on the decrease in the internal resistance of the encapsulated ZnO/CuO–MWCNTs nanostructured composites by the fabrication of multilayered structures, doping of ZnO and CuO, as well as the development of high-quality electrical contacts to the networks. However, it should be noted that to the best of our knowledge of literature, there is a minimal number of reports showing the performance of TEGs prepared from ZnO-based composites. The comparison of the performance of the demonstrated TEG with the available reports showed the performance comparable and even higher in terms of the demonstrated power density with similar state-of-the-art TEGs based on the composites fabricated from CNTs, ZnO, or inorganic thermoelectric materials as bismuth and antimony chalcogenides mixed with nonconductive environmentally neutral polymer matrixes, while using a dramatically less amount of CNTs, which makes these ZnO and CuO based composites more environmentally friendly (Table 3).

These results demonstrate that after further improving and optimizing the performance, the presented epoxy-encapsulated ZnO–MWCNT hybrid nanocomposites have a significant potential for domestic waste heat-to-power conversion applications.

## 4. Conclusions

Epoxy-encapsulated ZnO–MWCNT hybrid nanocomposites with MWCNT contents of 0.125, 0.25, and 0.5 wt.% showed a significantly enhanced thermoelectric performance compared to non-encapsulated ZnO–MWCNT hybrid nanostructured networks, as well as encapsulated ZnO nanostructured networks without MWCNTs. Nanocomposites were fabricated by the thermal oxidation of a self-assembling Zn nanostructured network spray-coated with a layer of MWCNTs. This was followed by encapsulation in commercially available adhesive using the drop-cast method. It was found that in non-encapsulated ZnO–MWCNT networks, the inclusion of MWCNTs resulted in a decrease in the Seebeck coefficient, which was attributed to the competing contribution of the p-type MWCNTs to the total Seebeck coefficient dominated by the n-type ZnO nanostructures. However, the encapsulation of the ZnO–MWCNT hybrid networks in epoxy adhesive resulted in an impressive increase in the absolute values of the Seebeck coefficient from ~100–180 μV·K^–1^ to ~570–900 μV·K^–1^. The increased Seebeck coefficient of the encapsulated ZnO–MWCNTs hybrid networks did not show a pronounced correlation with the content of MWCNTs, indicating that, in that case, the contribution of the MWCNTs was negligible. Simultaneously, the encapsulation of ZnO–MWCNTs resulted in a power-law decrease in their electrical resistance by 20–60 times depending on the MWCNT content, with the lowest decrease corresponding to the encapsulated ZnO nanostructured networks not containing MWCNTs, and highest decrease corresponding to the highest (0.5 wt.%) content of MWCNTs in the ZnO–MWCNT network. This effect was attributed to the formation of the MWCNT network during the encapsulation process, which contributed to the total conductance of the sample. Both the increase in the Seebeck coefficient and decrease in the resistance of ZnO and ZnO–MWCNTs networks after encapsulation in epoxy adhesive were attributed to the impact of the formed electrically conductive ZnO–epoxy interface layer, which simultaneously served as the charge trapping layer. This optimized the charge carrier concentration to achieve high Seebeck coefficient values. The calculated power factors of the encapsulated ZnO–MWCNTs hybrid networks exceeded the PF of these networks before encapsulation by 3–4 orders of magnitude. In addition, a two-leg thermoelectric generator composed from n-type epoxy-encapsulated ZnO–MWCNT hybrid nanocomposite and p-type polydimethylsiloxane-encapsulated CuO–MWCNT nanocomposite showed better performance at room temperature and 30 °C temperature difference compared to the similar devices. This illustrates the great potential of the developed epoxy-encapsulated ZnO–MWCNT hybrid nanocomposites for domestic waste heat-to-power conversion applications. In addition, methods for the further improvement of the developed nanocomposites were outlined.

## Figures and Tables

**Table 1:** Resistance of ZnO–MWCNT hybrid networks with different MWCNT wt.% before and after encapsulation in the epoxy adhesive.

MWCNT wt.% in the ZnO-MWCNT Hybrid Network	Resistance before Encapsulation, R_b_, ×10^6^ Ω	Resistance after Encapsulation, R_a_, ×10^6^ Ω	R_b_/R_a_
0	0.8 ± 0.02	0.044 ± 0.002	18
0.125	8.4 ± 0.2	0.368 ± 0.003	23
0.25	1.63 ± 0.03	0.059 ± 0.002	28
0.5	3.27 ± 0.03	0.054 ± 0.001	60

**Table 2:** Seebeck coefficient of ZnO and ZnO–MWCNT nanostructured networks before and after encapsulation in epoxy adhesive.

Nanostructured Network	MWCNT wt.%	Seebeck Coefficient before Encapsulation, S_b_, μV·K^–1^	Seebeck Coefficient after Encapsulation, S_a_ μV·K^–1^
ZnO, this work	0	−180 ± 15	−570 ± 50
ZnO–MWCNT, this work	0.125	−160 ± 10	−900 ± 100
ZnO–MWCNT, this work	0.25	−105 ± 10	−570 ± 40
ZnO–MWCNT, this work	0.5	−40 ± 5	−625 ± 55
ZnO [12]	0	−150 ± 40	-
Ni-CNTs/ZnO [35]	2.0	−260 ± 10	-
SWCNTs with 15 wt.% ZnO nanowires [34]	85	−24	-
ZnO: Al films deposited on MWCNT substrates [36,37]	0.1 g of MWCNTs	−130; −145	-
Zn_0.98_Al_0.02_O mixed with MWCNTs [38]	0.1	−80	-
Epoxy–MWCNT-TiO_2_ [50]	6–8	-	−15–25

**Table 3:** Comparison of maximal thermally generated open-circuit voltage (U_0_) and output power density (Pd) per one degree Kelvin and per one leg pair for different types of nonconductive polymer-based thermoelectric generators.

Type of the Thermoelectric Generator	CNT wt.%	U_0_, mV·K^–1^	Pd, nW·cm^–2^·K^–1^
Epoxy-encapsulated ZnO–MWCNT-PDMS-encapsulated CuO–MWCNT (with 0.9 wt.% MWCNT), this work	0.5	0.13	7.5
SWCNT-ZnO (n- and p-doped) [34]	85	0.03	-
ZnO thin films grown on polyimide substrates [40]	-	0.07	6
Mixed in PVA MWCNT-Sb_2_Te_3_ and MWCNT-Bi_2_Se_3_ [53]	25–30	0.07–0.14	0.06–0.13
One-type legs PVA/Bi_2_Te_3_ [54]	-	0.1	0.02
One-type legs MWCNT/PDMS [55]	-	0.002	-
Sb_2_Te_3_-Bi_2_Te_3_ thin films sputtered on the flexible substrate [56]	-	0.1	0.04
Bi_2_Te_3_-based printed wearable TEG [57]	-	4	3.4

## Data Availability

The data presented in this study are available upon request from the corresponding author. The data are not publicly available as they are part of ongoing research.

## Supplementary Materials

The following supporting information can be downloaded at: https://www.mdpi.com/article/10.3390/polym15234540/s1, Figure S1: Current-voltage curves of ZnO network not containing MWCNTs before (black curve) and after (red curve) encapsulation in epoxy adhesive; Figure S2: Current-voltage curves of ZnO-MWCNT network containing 0.125 wt.% of MWCNTs before (black curve) and after (red curve) encapsulation in epoxy adhesive; Figure S3: Current-voltage curves of ZnO-MWCNT network containing 0.5 wt.% of MWCNTs before (black curve) and after (red curve) encapsulation in epoxy adhesive.
