# Supplementary material for: Epoxy-Encapsulated ZnO–MWCNT Hybrid Nanocomposites with Enhanced Thermoelectric Performance for Low-Grade Heat-to-Power Conversion
Source: Polymers (Basel). 2023 Nov 26;15(23):4540. doi: 10.3390/polym15234540 (PMC10708207; doi:10.3390/polym15234540)
Supplement: Supplementary file 1 [file polymers-15-04540-s001.zip › polymers-2702684-supplementary.pdf]

Supplementary material

Epoxy-encapsulated ZnO-MWCNT hybrid nanocomposites with enhanced thermoelectric performance for low-grade heat-to-power conversion

Margarita Volkova[1], Raitis Sondors[1], Elmars Spalva[2], Lasma Bugovecka[1], Artis Kons[3], Raimonds Meija[2], Jana Andzane[1*]

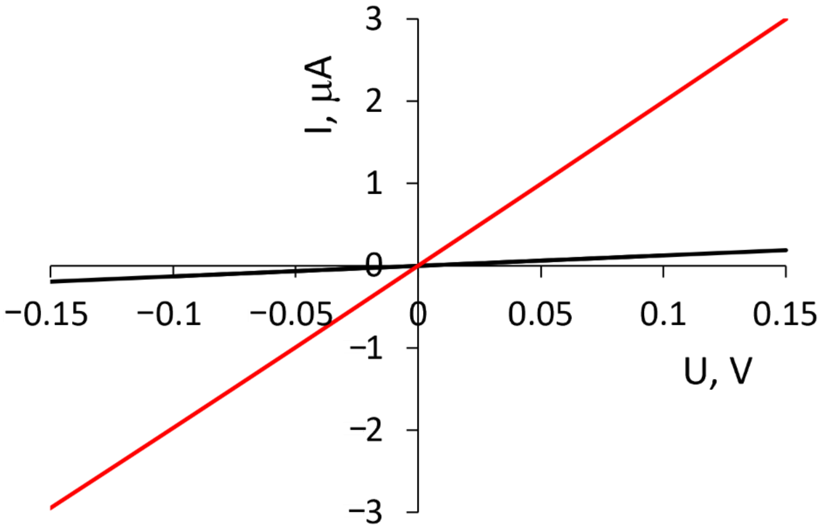

Figure S1. Current-voltage curves of ZnO network not containing MWCNTs before (black curve) and after (red curve) encapsulation in epoxy adhesive.

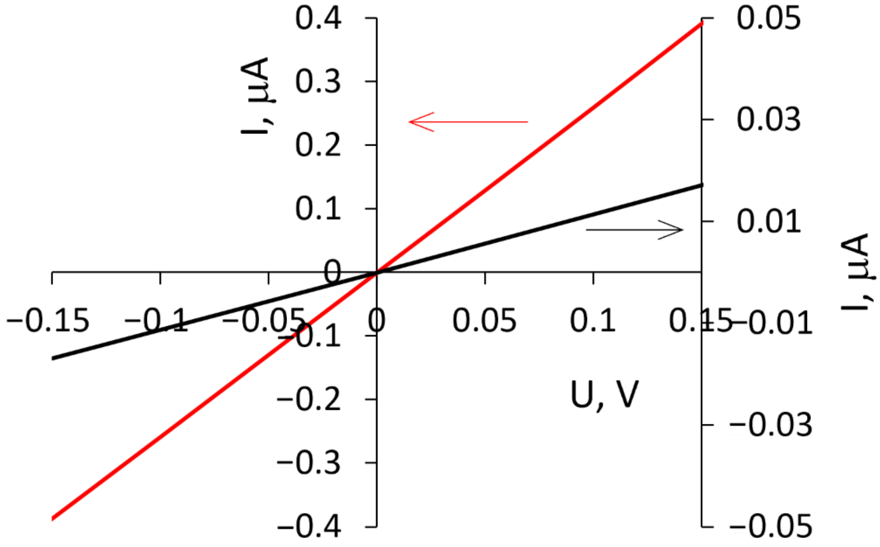

Figure S2. Current-voltage curves of ZnO-MWCNT network containing 0.125 wt.% of MWCNTs before (black curve) and after (red curve) encapsulation in epoxy adhesive.

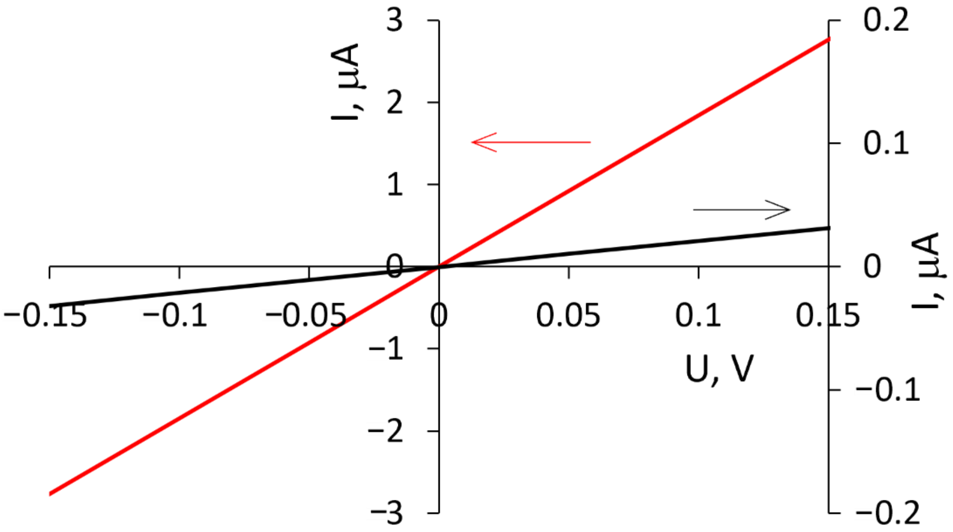

Figure S3. Current-voltage curves of ZnO-MWCNT network containing 0.5 wt.% of MWCNTs before (black curve) and after (red curve) encapsulation in epoxy adhesive.
